# Deep learning classification of uveal melanoma based on histopathological images and identification of a novel indicator for prognosis of patients

**DOI:** 10.1186/s12575-023-00207-0

**Published:** 2023-06-02

**Authors:** Qi Wan, Xiang Ren, Ran Wei, Shali Yue, Lixiang Wang, Hongbo Yin, Jing Tang, Ming Zhang, Ke Ma, Ying-ping Deng

**Affiliations:** Department of Ophthalmology, West China Hospital, Sichuan University, Chengdu City, Sichuan Province China

**Keywords:** Deep learning, Histopathological images, Prognosis, Uveal melanoma, Subtype

## Abstract

**Background:**

Deep learning has been extensively used in digital histopathology. The purpose of this study was to test deep learning (DL) algorithms for predicting the vital status of whole-slide image (WSI) of uveal melanoma (UM).

**Methods:**

We developed a deep learning model (Google-net) to predict the vital status of UM patients from histopathological images in TCGA-UVM cohort and validated it in an internal cohort. The histopathological DL features extracted from the model and then were applied to classify UM patients into two subtypes. The differences between two subtypes in clinical outcomes, tumor mutation, and microenvironment, and probability of drug therapeutic response were investigated further.

**Results:**

We observed that the developed DL model can achieve a high accuracy of >  = 90% for patches and WSIs prediction. Using 14 histopathological DL features, we successfully classified UM patients into Cluster1 and Cluster2 subtypes. Compared to Cluster2, patients in the Cluster1 subtype have a poor survival outcome, increased expression levels of immune-checkpoint genes, higher immune-infiltration of CD8 + T cell and CD4 + T cells, and more sensitivity to anti-PD-1 therapy. Besides, we established and verified prognostic histopathological DL-signature and gene-signature which outperformed the traditional clinical features. Finally, a well-performed nomogram combining the DL-signature and gene-signature was constructed to predict the mortality of UM patients.

**Conclusions:**

Our findings suggest that DL model can accurately predict vital status in UM patents just using histopathological images. We found out two subgroups based on histopathological DL features, which may in favor of immunotherapy and chemotherapy. Finally, a well-performing nomogram that combines DL-signature and gene-signature was constructed to give a more straightforward and reliable prognosis for UM patients in treatment and management.

**Supplementary Information:**

The online version contains supplementary material available at 10.1186/s12575-023-00207-0.

## Introduction

Although less frequent than cutaneous melanoma, uveal melanoma (UM) is the most prevalent type of primary malignancy in adult eyes. UM occurs for just 5% of all melanomas, but it causes 13% of melanoma-related deaths [1]. The prevalence of UM has been connected to a number of parameters like age, race, iris color, and so on [2, 3]. Despite treatment for UM at the primary stage, approximately half of the patients have metastases, with the liver being the most frequent metastatic location. After metastasis, the median survival time is decreased to 1 year [4, 5]. Despite the fact that various eye-sparing therapies, such as radiation, transpupillary thermotherapy, and photodynamic therapy, have emerged in recent years, their outcomes are not always ideal due to challenges in understanding their pathophysiology.

The prognosis of UM, like that of many other types of tumors, is highly reliant on timely detection and treatment [6]. With the rising practical application of new therapeutic strategies, current research is mostly focused on the discovery of novel prognostic signatures, which are commonly utilized to assess the risk of certain cancers and early diagnosis [7, 8]. Prognostic indicators, such as the patient's age, tumor location, size, and tumor histopathological and genetic characteristics, are all important factors for primary UM [9]. The use of prognostic indicators in clinical settings can help to steer risk patients through specific therapy and care, and perhaps avert life-threatening metastases [10–12]. For example, prior studies struggled to identify relevant mRNAs, microRNAs, or DNA methylation combinations as biomarkers to predict UM survival using bioinformatics [13–15]. In terms of UM cell morphological types, UM can be classified into three subtypes: epithelioid, spindle, and mixed cell types. The epithelioid cells type accounts for roughly 3–5% of all UM and is linked with the worst outcome. Spindle cell type comprises over 40% of all UM and is associated with better prognosis [16, 17]. The identification of histologic types in UM is critical for tumor prognosis and therapy [18]. This emphasizes the critical need for innovative strategies to classify UM subtypes that are consistently related to prognosis.

Furthermore, pathology procedures frequently entail arduous and time-consuming stages that can result in mistakes and negatively impact healthcare [18]. Recent advances in artificial intelligence have produced exceptional performances on diagnostic and prognostic tasks [19]. Deep learning, for example, has been widely employed in digital histopathology for applications such as cancer classification, cell identification, and patient outcome stratification in whole-slide images (WSIs) [20–23]. With the advantage of deep learning and the availability of large numbers of histology slides, there is a novel chance to reassess traditional techniques to predict the diagnosis and prognosis of patients [24, 25]. However, this technique is often hard to interpret. To solve these restrictions, we combined transcriptome datasets and employed bioinformatic analysis to explore the pathogenic mechanism on a genomic scale.

In this work, we created a deep learning model to predict the vital status of UM from histopathological images. Based on deep learning features, UM patients were successfully classified into two subtypes with distinct molecular characteristics and survival outcomes. According to histopathological classification, we constructed a histopathologic deep learning-signature, gene-signature as well as nomogram which might guide the prognosis and immunotherapy prediction of UM patients.

## Materials and methods

### UM cohort collection

**Fig. 1:**
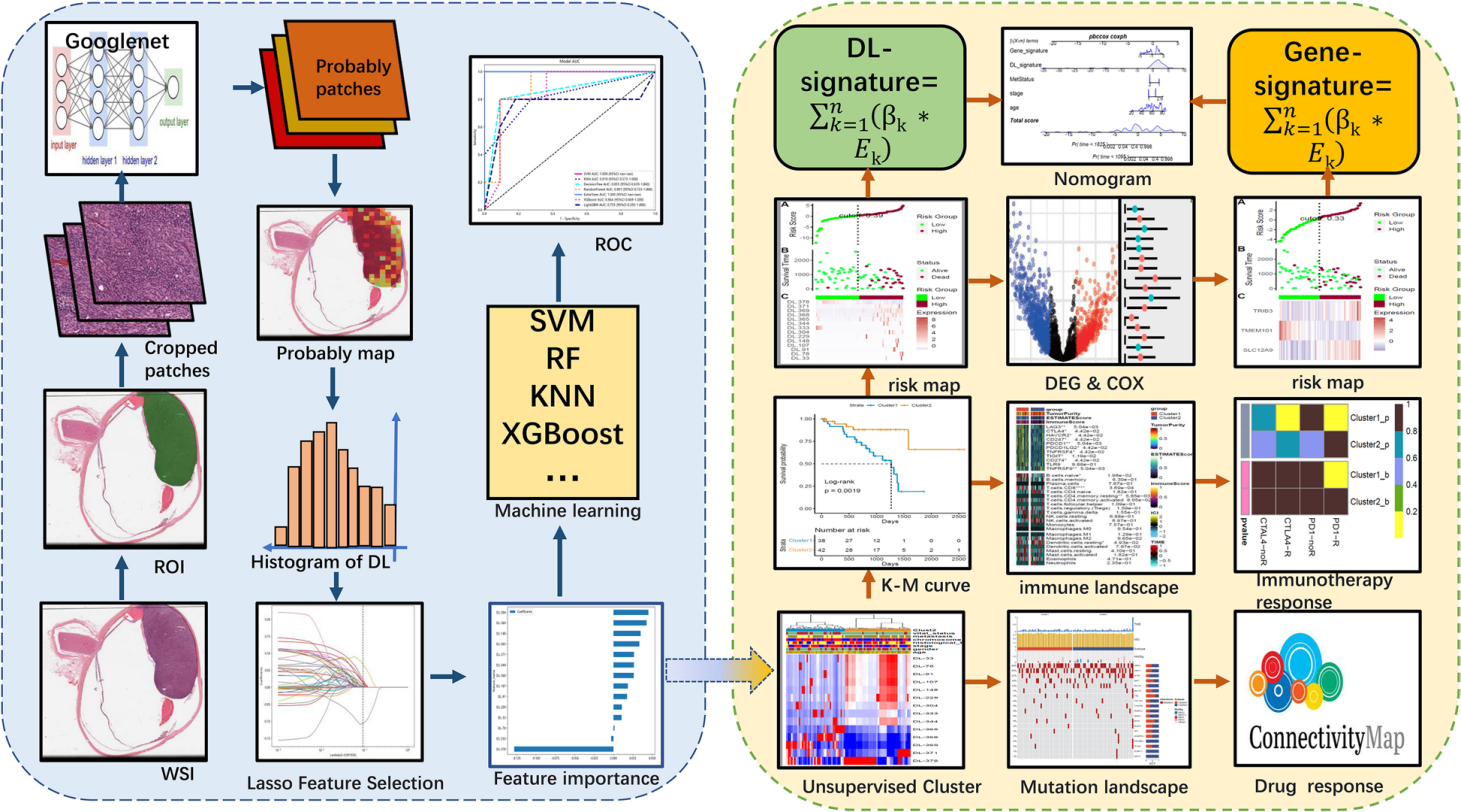
The simplified procedure for the current study

In this work, we first acquired 80 whole-slide images (WSIs) of UM from TCGA-UVM cohort, deposited in The Cancer Genome Atlas database, which was further assigned two labels: alive or dead status. The paired RNA-seq of 80 UM samples were utilized to explore the potential gene signature by bioinformatic analysis. Besides, the outside UM cohorts were retrieved from open-access resources (Gene Expression Omnibus and ArrayExpress databases) and internal dataset. The following strategies were used to find appropriate cohorts: 1) the sample belongs to a human being; 2) the cohort included survival data; 3) the cohort contained RNA-seq or whole-slide images of Hematoxylin and Eosin (H&E) staining; and 4) these cohorts were derived from several separate studies. According to the selection criterion, four open-accessed UM cohorts (GSE22138, GSE27831, GSE84976, and E-MTAB-4097) and one internal UM cohort (HX cohort) were included in our study. The internal UM cohort (67 samples) was consecutively recruited by the Ophthalmology department of West China Hospital, Chengdu, China from 2009 to 2016. The simplified procedure for the current study was displayed in Fig. 1.

### WSIs annotation and processing

To largely avoid the effect of irrelevant areas and reduce the effort of the classification system, professional pathologists manually marked regions of uveal melanoma (ROI) on WSIs by using the following guidelines: (1) the cancer cells should comprise more than 80% of ROI and (2) evident interfering features such as creases, hemorrhage, necrosis, and hazy regions should be avoided. Qupath (v.0.2.3) was used to conduct the annotation. Considering the extraordinarily huge picture size of WSIs (usually 100,000*80,000 pixels), the WSIs were cropped into numerous patches. Next, patches with more than 50% overlap of the melanoma ROI were selected for subsequently analyze. The WSIs generally contained a number of patches ranging from 325 to 2633 in TCGA-UVM. The TCGA-UVM cohort was randomly classified into separate train and validation datasets (7:3 ratio) (Table 1). The train dataset was employed for model development and hyperparameter tuning, meanwhile the validation dataset and internal UM cohort were utilized to assess generalization performance. For training patches, both data augmentation and normalization were used, however just normalization was used for validated patches. Random affine transformation and horizontal flipping of patches were employed in our study for data augmentations. The enhanced patches were center cropped to 224 * 224 pixels after z-score normalization on RGB channels.

**Table 1:** Clinical information of train and validation datasets in TCGA_UVM cohort and HX cohort. IQR means interquartile range

		Datasets				
	level	train	validation	HX cohort	p	test
n		56	24	67		
futime (median [IQR])		759.50 [466.25, 1159.75]	816.00 [352.00, 1290.25]	1590.00 [555.00, 2280.00]	1.93E-04	Kruskal–Wallis Test
fustat (median [IQR])		0.00 [0.00, 1.00]	0.00 [0.00, 1.00]	0.00 [0.00, 0.00]	9.57E-01	Kruskal–Wallis Test
age (median [IQR])		60.00 [49.75, 74.00]	64.00 [54.00, 76.00]	52.00 [41.00, 59.00]	2.89E-05	Kruskal–Wallis Test
gender (%)	female	24 (42.9)	11 (45.8)	31 (46.3)	9.26E-01	Chisq Test
	male	32 (57.1)	13 (54.2)	36 (53.7)		
stage (%)	Stage II	26 (46.4)	10 (41.7)	—	3.21E-01	Chisq Test
	Stage III	26 (46.4)	14 (58.3)	—		
	Stage IV	4 (7.1)	0 (0.0)	—		
histological_type (%)	epithelioid	9 (16.1)	4 (16.7)	25 (37.3)	5.48E-03	Chisq Test
	mixed	27 (48.2)	10 (41.7)	13 (19.4)		
	spindle	20 (35.7)	10 (41.7)	29 (43.3)		
chromosome.3.status (%)	disomy	25 (44.6)	13 (54.2)	—	5.91E-01	Chisq Test
	monosomy	31 (55.4)	11 (45.8)	—		
MetStatus (%)	Metastatic	20 (35.7)	6 (25.0)	18 (26.9)	4.79E-01	Chisq Test
	Non-metastatic	36 (64.3)	18 (75.0)	49 (73.1)		
vital_status (%)	alive	40 (71.4)	17 (70.8)	51 (76.1)	8.00E-01	Chisq Test
	dead	16 (28.6)	7 (29.2)	16 (23.9)		
SCNA_Cluster (%)	A	8 (14.3)	7 (29.2)	—	2.78E-01	Chisq Test
	B	17 (30.4)	6 (25.0)	—		
	C	18 (32.1)	4 (16.7)	—		
	D	13 (23.2)	7 (29.2)	—		

### Deep-learning (DL) feature extraction and selection

The WSIs correlated with UM in TCGA-UVM and HX cohorts were firstly cropped into patches without overlap. We subsequently performed a weakly supervised method to trained a Google-net model for 50 epochs by using WSI-level labels for supervision. The optimizer was SGD with a learning rate of 10–2 and L2 regularization of 10–5. Then, this classifier (Google-net model) was used to label all the patches in the WSI and store the labels in a heatmap, representing the probability score of each patch. Due to the WSIs contained numerous patches, we assembled the probably patches into a probably heatmap of WSI, which was then applied to estimate the histopathologic DL features based on histogram of patch likelihood. The histopathological DL features were defined as the structures data come from histogram of patch likelihood. For removing redundant DL features, Pearson correlation analysis was firstly performed. If the coefficient of two features was larger than 0.9, one of the feature will be eliminated. Following that, to identify important DL features, we split the histopathological DL features in TCGA-UVM cohort into training and testing sets and standardized the data. Next, we trained a Lasso regression model on the training dataset. When using Lasso for feature selection, we first need to select an optimal regularization parameter value and the corresponding L1 penalty coefficient. We further select the features with coefficients > 0 and assess the importance of each feature based on its weight coefficients in the Lasso model. The larger the parameter estimate (absolute value), the higher the importance of that feature. Finally, retrain the model using the selected feature subset and evaluate the performance on the testing set. Based on these feature vectors, 7 traditional machine learning classifiers (SVM, KNN, Decision-Tree, Random-Forest, Extra-Tree, XGBoost, and LightGBM) were then trained to predict the vital status for each WSI.

### Unsupervised cluster of DL features

To perform unsupervised clustering, the identified important DL features from TCGA-UVM cohort were first extracted. Next, hierarchical clustering in the "ClassDiscovery" package was to identify a potentially relevant subtype of UM. To compare the prognosis of subgroups defined by DL features, Kaplan–Meier (K-M) curves were used. Besides, to discover the underlined cancer hallmark pathways associated with a subtype of UM, we conducted gene set variation analysis (GSVA) method to assess the pathway activities using Cancer Hallmark set (h.all.v7.0.symbols) in MSigDB (https://www.gsea-msigdb.org).

### Landscape of mutated and immune characteristics in UM subtype

Two methods were usually used for decoding the immune microenvironment (ESTIMATE and CIBERSORT). The "ESTIMATE" approach was generally applied to estimate the total infiltrated immune score, stromal score, estimate score, and tumor purity in tumor tissue. The "CIBERSORT" approach was used to quantify the proportion of 22 distinct kinds of immune cells in the UM tumor microenvironment based on 1,000 permutations of the LM22 signature. We initially used the ESTIMATE and CIBERSORT approach to examine the immune score, estimate score and tumor purity, and relative percentages of 22 different kinds of immune cells in UVM patients. The different immune microenvironment characteristics and expression of immune checkpoint genes between UM subtypes were subsequently investigated. Moreover, UM patients, in particular, may benefit from subtype-specific mutations as a therapy target. As a result, we compared the mutational frequency, Microsatellite instability (MSI), mutation burden (TMB), and mutated signatures between UM subtypes. Eventually, a DL-signature was developed by Multivariate Cox modeling, and DL-signature associated scores for each patient were produced by the formula: ${\sum }_{{\text{k}}= \text{1} }^{\text{n}}\left({\beta }_{k}\text{* }{\text{E}}{\text{k}}\right)$.

### Immunotherapy response and potential drugs prediction

To test distinct immunotherapy responses between UM subtypes, three previous melanoma cohorts including the Chen et al. study [26], Prat A et al. study [27], and Hugo et al. study [28], which treated with anti-CTLA-4 or anti-PD-1 treatment was acquired and analyzed. The baseline features of the three cohorts were listed in Table S1. These cohorts' gene expression patterns, as well as immunotherapy response data, were collected from published research. To predict the response of immunotherapy in UM subtypes, Subclass Mapping (SubMap) analyses were used to assess the gene expression similarity between the UM subtypes (Cluster 1 and Cluster 2) and the previous melanoma patients with various anti-CTLA-4 and anti-PD-1 therapeutic responses. Furthermore, in comparison with gene expression patterns of drugs collected from the Connectivity Map, we can systematically compute a therapeutic score. Drugs with significantly lower scores, the more likely this drug is to reverse the molecular features of the disease, and hence may suggest potential therapeutic possibilities [29].

### Identification and validation of histopathologic gene-signature

Firstly, the ‘Limma’ package in R software was employed to identify differentially expressed genes (DEGs) between UM subgroups. The selection of survival-related DEGs were subjected to univariate Cox regression analysis. In addition, we performed 1000 iterations of Lasso-penalized analysis to narrow down the list of important histopathologic genes. Combined with the AUCs of gene combinations, we finally select the candidate gene signatures. Multivariate Cox modeling was used to create a gene-signature, and gene-signature correlated risk score for each patient was produced by the formula: ${\sum }_{{\text{k}}= \text{1} }^{\text{n}}\left({\beta }_{k}\text{* }{\text{E}}{\text{k}}\right)$. UM Patients' risk scores were individually assessed in five independent UM cohorts (TCGA-UVM, GSE27831, GSE22138, GSE84976, and E-MTAB-4097). The median risk score was employed to categorize patients as high-risk or low-risk. The K-M curves were drawn and log-rank tests were used to examine the different significance in survival outcomes between the two groups. The AUC value calculated by the time-dependent receiver operating characteristic (ROC) curve was used to assess the prediction performance of the histopathologic gene-signature in the prognostic model. Furthermore, the concordance index (C-index) was conducted to assess the predictive power of the DL-signature, histopathologic gene-signature, and conventional clinical factors.

### Statistical analysis

R (version 4.0.3) or Python (version 3.8.0) with installed packages were used for all statistical studies. All deep learning frameworks were implemented via Pytorch (version 1.10.1) in Python on an Nvidia GeForce RTX-3080 GPU workstation with 10 GB of memory. Python's "sklearn" package was used to run the machine learning algorithms. The "survival" and “survivalROC” packages in R were used to perform K-M and ROC curves. The “ClassDiscovery” package in R was used to conduct unsupervised clustering. “CIBERSORT” and “estimate” packages in R were employed to assess the immune microenvironment. The Pearson coefficients were used to evaluate the correlation test. In comparison with more than two groups, the Kruskal–Wallis test was employed, whereas the Wilcoxon test was used for comparison between the two groups. The chi-square test was used to investigate the relationships between subgroups and clinicopathological features. The Cox regression analysis yielded hazard ratios (HRs) and 95% confidence intervals (CIs). All statistical tests were considered significant with a *p* < 0.05.

## Results

### Performance of the histopathological classifier

The histopathological classifier was developed in the train dataset, and then verified in the validation dataset and HX cohort, which contained two successive steps: patches prediction and WSIs prediction. To summarize, WSIs were firstly annotated to identify the tumor area (ROI). The ROI was cropped into patches, which were then input into a deep learning network (Google-net) to predict vital status at the level of patches. Second, a histogram of patches likelihood was used to integrate multiple probable patches into a whole probably heatmap of WSI. Eventually, we applied multiple machine learnings to predict the UM patient's vital status.

**Fig. 2:**
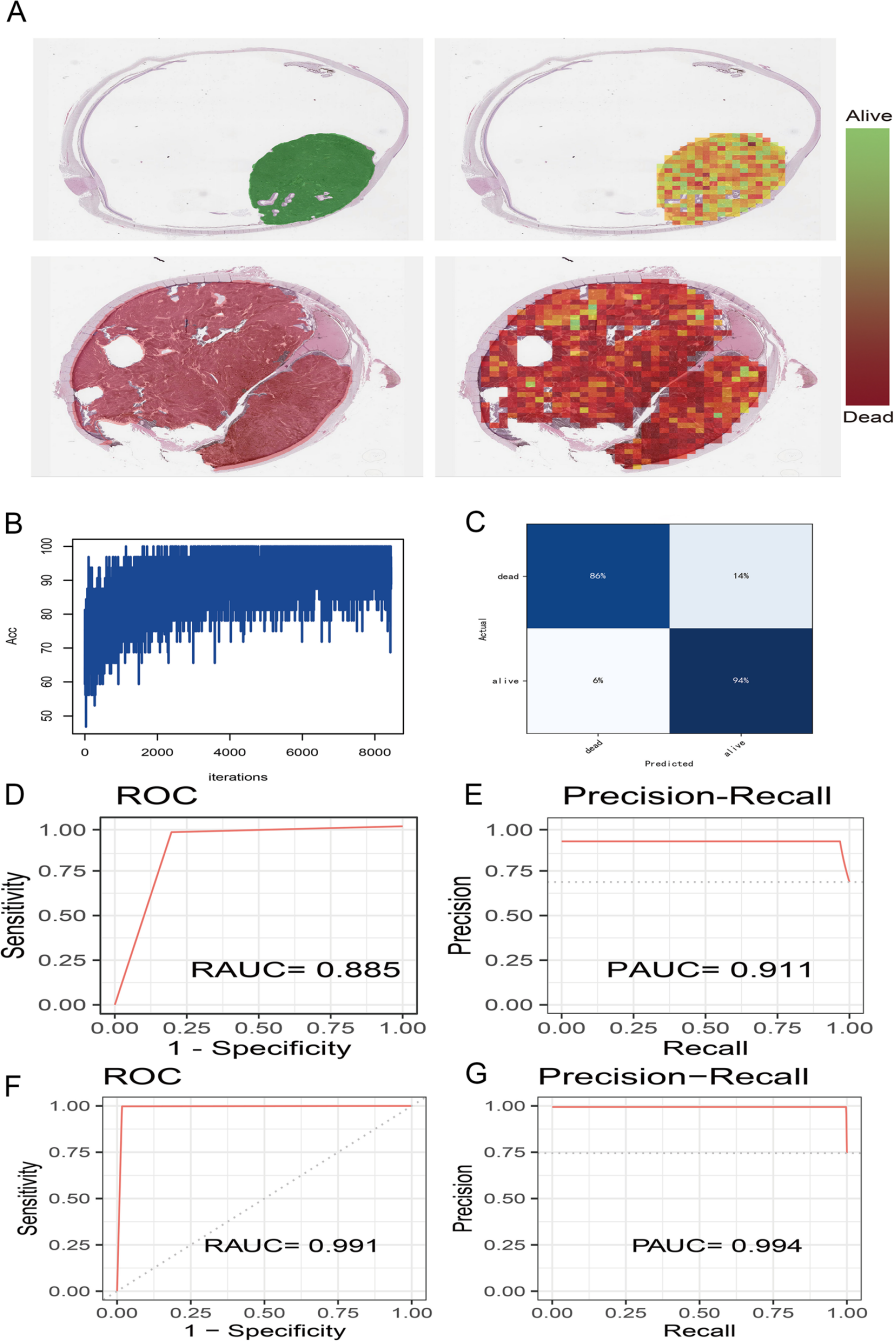
The deep learning (Google-net) model for patches prediction. **A** Probably heatmaps of alive and dead status at the stage of patches prediction. The color bars represent the vital status probability of each patch. **B** The accuracy curve of deep learning model training. **C** The confusion matrix of deep learning model. **D** The ROC curve and AUC value (RAUC) of deep learning model in TCGA-UVM cohort. **E** The Precision-Recall curve and AUC value (PAUC) of deep learning model in TCGA-UVM cohort. **F** The RAUC of deep learning model in HX cohort. **G** The PAUC of deep learning model in HX cohort

The performance of the histopathological classifier was evaluated using the TCGA-UVM validation dataset. Two typical probably heatmaps that respectively forecast patch levels for dead and alive status (Fig. 2A). As the number of training iterations increases, the training accuracy converges near 90% at the first 2000 iterations (Fig. 2B). The confusion matrix illustrated that the Google-net model achieved a high accuracy of 90% (Fig. 2C). Besides, the ROC curve and Precision-Recall curve showed that our model performed a RAUC of 0.885 (Fig. 2D) and a PAUC of 0.911 (Fig. 2E). In the HX cohort, our model also performed well with a RAUC of 0.991 (Fig. 2F) and a PAUC of 0.994 (Fig. 2G).

**Fig. 3:**
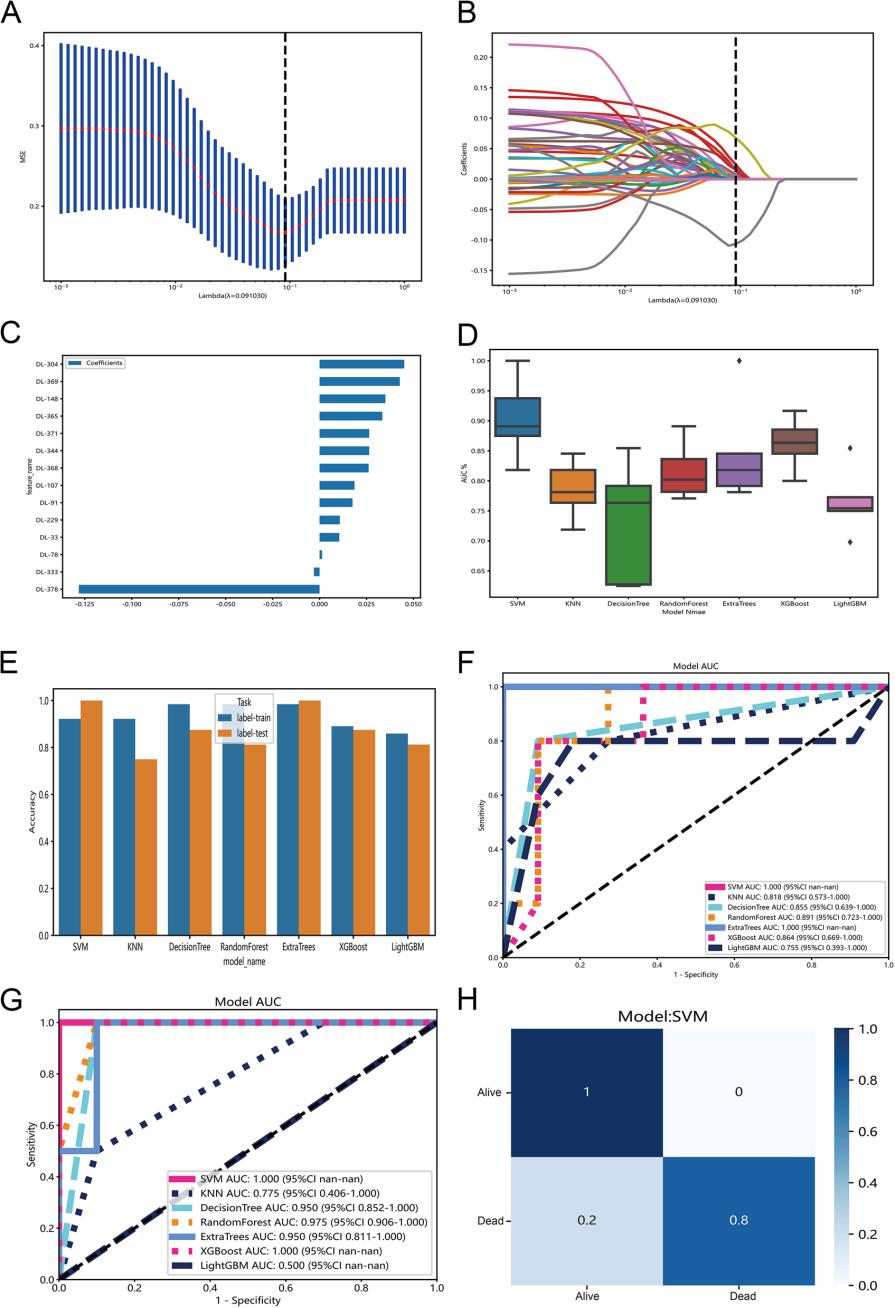
The deep learning features selection. **A** The distribution of the lowest mean squared error (MSE) with the corresponding penalization lambda value in LASSO-penalized model. **B** LASSO coefficient profiles of all deep learning features and the selected criterion of lambda. **C** Feature weight bar chart for the LASSO-penalized model. **D** The AUCs distribution of seven machine learning classifiers (SVM, KNN, Decision-Tree, Random-Forest, Extra-Tree, XGBoost, and LightGBM) with tenfold cross-validation. **E** The accuracy distribution of seven machine learning classifiers in train and test datasets. **F** The ROC curves of 7 machine learnings in TCGA-UVM cohort. **G** The ROC curves of 7 machine learnings in HX cohort. **H** The confusion matrix of SVM classifier in TCGA-UVM cohort

Subsequently, we extracted 379 deep learning (DL) features from the histogram of patches likelihood (Table S2). After the Pearson correlation analysis, 268 DL features were retained for LASSO-penalized feature selection. When the penalization lambda is 0.091, we found the LASSO model has the lowest mean squared error (MSE) (Fig. 3A). Based on the selected criterion of lambda, there were 14 DL features with a coefficient > 0 (Fig. 3B). Finally, the LASSO-penalized model identified 14 DL features, and the relative importance of DL features were illustrated in Fig. 3C. Afterwards, the 14 DL features were put into seven traditional machine learning classifiers with tenfold cross-validation. The AUCs distribution of seven machine learning classifiers suggested that the SVM classifier has the highest AUCs (Fig. 3D). The accuracy of different machine learning methods in train and test datasets was manifested in Fig. 3E. The ROC curves indicated that the AUCs of SVM and Extra-Trees classifiers achieved 1 (Fig. 3F). The model was also validated in the HX cohort and the result revealed that the AUCs of SVM and Extra-Trees classifiers were 1 and 0.95, respectively (Fig. 3G). The confusion matrix of the SVM classifier was shown in Fig. 3H. The detailed parameters (Accuracy, AUC, Sensitivity, Specificity, PPV, NPV, Precision, and Recall) for the assessment of models were listed in Table S3.

### Unsupervised cluster of DL features

**Fig. 4:**
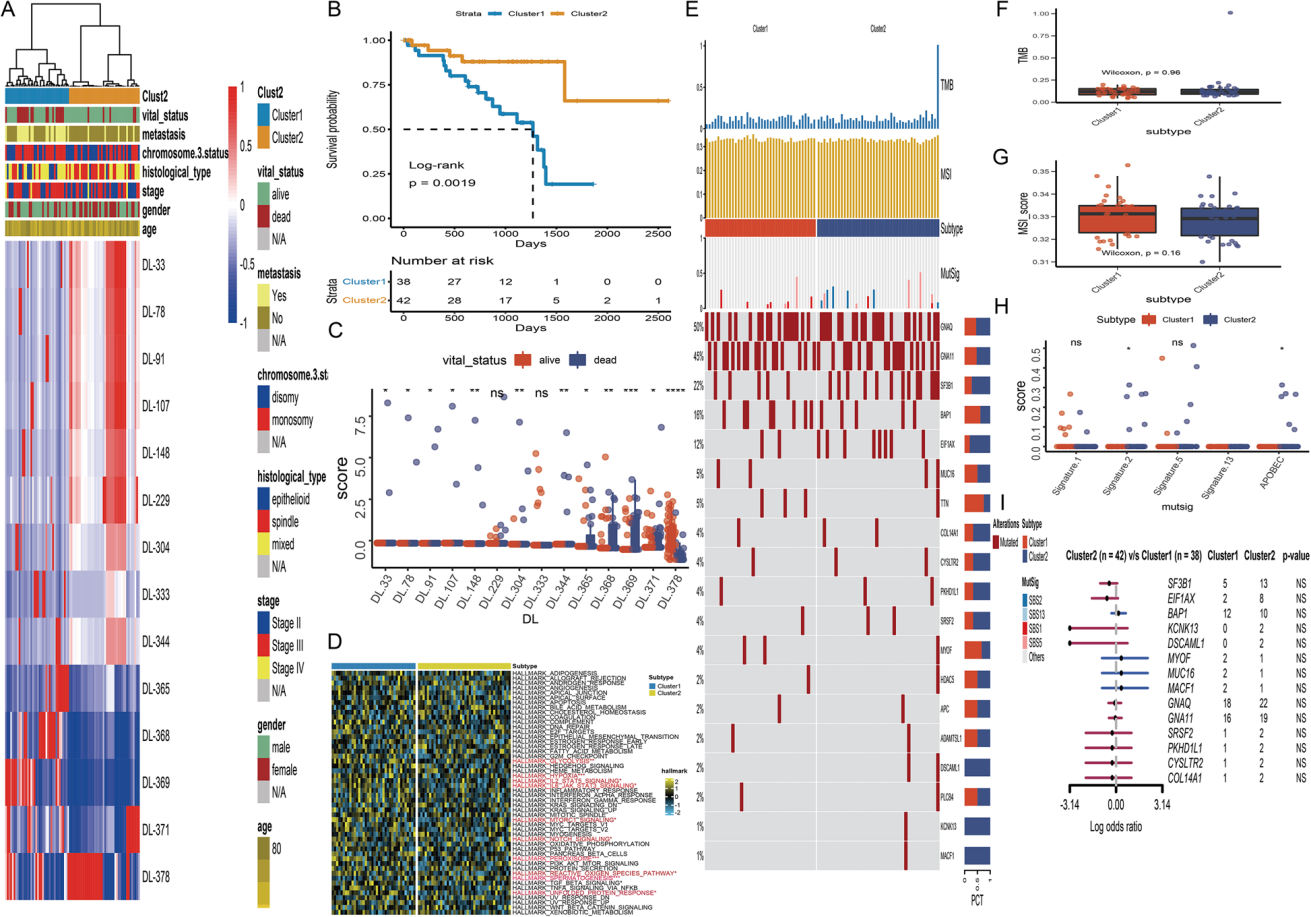
Unsupervised clustering of deep learning features in TCGA-UVM cohort. **A** Comprehensive heatmap with unsupervised clustering showed distinctive characteristics of deep learning features and clinical characteristics. **B** Kaplan–Meier curve of survival probability between Cluster1 and Cluster2 subtype. **C** Boxplot of the 14 deep learning features between alive and dead status. **D** Heatmap of 50 cancer Hallmark pathways between Cluster1 and Cluster2 subtypes. **E** Comprehensive OncoPrint plots of mutated characteristics in Cluster1 and Cluster2 subtypes. **F** Boxplot of TMB between Cluster1 and Cluster2 subtypes. **G** Boxplot of MSI between Cluster1 and Cluster2 subtypes. **H** The distributions of mutational signatures in Cluster1 and Cluster2 subtypes. **I** Forest plots of the top mutated genes between Cluster1 and Cluster2 subtypes

The 14 DL features were further conducted to investigate the key clusters in the TCGA-UVM cohort. By using unsupervised clustering (k = 2), we were able to identify two stable subtypes: Cluster1 (38 UM patients) and Cluster2 (52 UM patients). A comprehensive heatmap was created to show the link between subtypes and clinical features (Fig. 4A). The chi-square tests determined significant differences in metastasis, histological type, and vital status between subtypes (Table 2). Furthermore, K-M curves demonstrated that UM patients in Cluster1 have a lower survival probability than the Cluster2 subtype, following log-rank test *p* = 0.0019 (Fig. 4B). The boxplot uncovered that almost all of the 14 DL features (apart from DL-229 and DL-333) were significantly different distributed between alive and dead status (Fig. 4C). GSVA was used to examine biochemical pathways shared by distinct Cluster1/Cluster2 subtypes. A heatmap of 50 cancer Hallmark pathways was visualized to explore the different expressions between Cluster1 and Cluster2 subtypes (Fig. 4D). The Wilcoxon tests discovered that 10 cancer Hallmark pathways were differentially expressed between two subtypes, which included glycolysis, hypoxia, IL2-STAT5-signaling, ILl6-JAK-STAT5 signaling, MTORC1 signaling, notch signaling, peroxisome, reactive oxygen species pathway, spermatogenesis, and unfolded protein response.

**Table 2:** Clinical features of subtypes in TCGA_UVM cohort. IQR means interquartile range

	level	Cluster1	Cluster2	p	test
n		38	42		
futime (median [IQR])		814.50 [458.75, 1161.25]	759.50 [433.50, 1204.25]	9.96E-01	wilcox Test
fustat (median [IQR])		0.00 [0.00, 1.00]	0.00 [0.00, 0.00]	5.05E-04	wilcox Test
age (median [IQR])		60.00 [53.00, 71.75]	63.50 [51.00, 75.00]	8.25E-01	wilcox Test
gender (%)	female	13 (34.2)	22 (52.4)	1.58E-01	Chisq Test
	male	25 (65.8)	20 (47.6)		
stage (%)	Stage II	13 (34.2)	23 (54.8)	1.75E-01	Chisq Test
	Stage III	23 (60.5)	17 (40.5)		
	Stage IV	2 (5.3)	2 (4.8)		
histological_type (%)	epithelioid	10 (26.3)	3 (7.1)	5.60E-03	Chisq Test
	mixed	20 (52.6)	17 (40.5)		
	spindle	8 (21.1)	22 (52.4)		
chromosome.3.status (%)	disomy	14 (36.8)	24 (57.1)	1.12E-01	Chisq Test
	monosomy	24 (63.2)	18 (42.9)		
MetStatus (%)	Metastatic	21 (55.3)	5 (11.9)	9.79E-05	Chisq Test
	Non-metastatic	17 (44.7)	37 (88.1)		
vital_status (%)	alive	20 (52.6)	37 (88.1)	1.14E-03	Chisq Test
	dead	18 (47.4)	5 (11.9)		
SCNA_Cluster (%)	A	4 (10.5)	11 (26.2)	7.66E-02	Chisq Test
	B	10 (26.3)	13 (31.0)		
	C	10 (26.3)	12 (28.6)		
	D	14 (36.8)	6 (14.3)		

### Landscape of mutated and immune characteristics in UM subtype

To detect subtype-specific mutations in UM, the "maftools" package in R software was initially used to generate oncoPrint plots of the top popular mutant genes in TCGA-UVM cohort, which contained GNAQ (50%), GNA11 (45%), SF3B1 (22%), BAP1 (16%), EIF1AX (12%) … (Fig. 4E). Among the frequently mutated genes, we observed that Cluster2 enriched more mutations of SF3B1(Cluster2: Cluster1 = 13:5), and EIF1AX (Cluster2: Cluster1 = 8:2), while Cluster1 subtype harbored more mutations of (Cluster2: Cluster1 = 10:12). However, the forest plot revealed that these mutated genes have no significant differences between Cluster2 and Cluster1subtypes (Fig. 4I). Besides, Tumor mutation burden (TMB) and Microsatellite instability (MSI) have emerged as promising biomarkers for the prediction of various tumor types, prognosis, and treatment response. As a consequence, TMB (Fig. 4F) and MSI (Fig. 4G) were compared between two subtypes, and the Wilcoxon tests found that there were no significant differences. According to recent tumor genomics research, the APOBEC signature is one of the most prominent mutational signatures in tumors. In addition, Other top mutational signatures in primary tumors such as signature 1 (age-related), signature 2 (APOBEC-mediated activities), and signature 13 (APOBEC-mediated processes) were also included in our analyses. The box plot indicated that only signature 2 and APOBEC signature have significant differences between Cluster2 and Cluster1 subtypes (Fig. 4H).

**Fig. 5:**
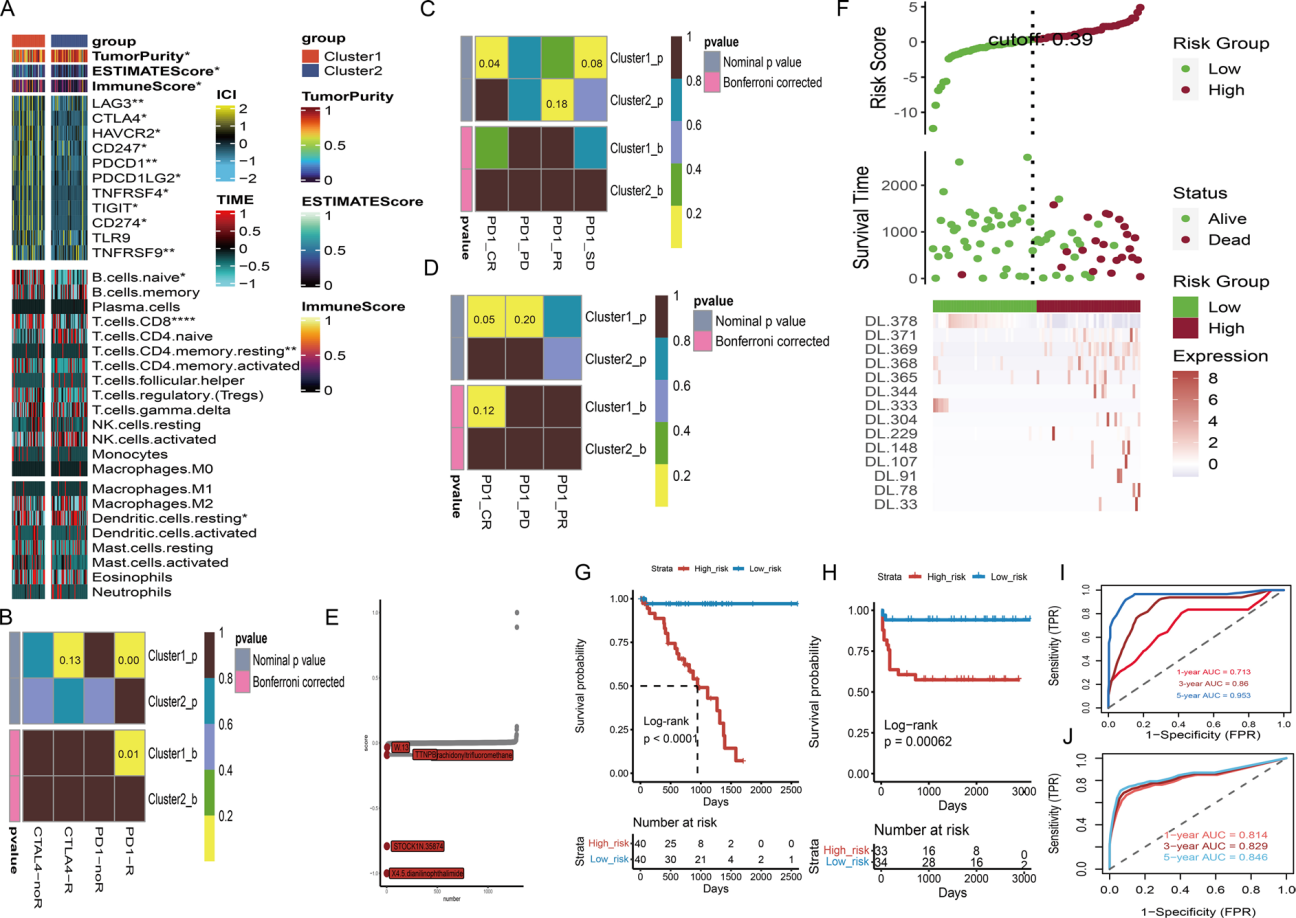
Subtype-specific immunophenotype and therapeutic prediction. **A** Comprehensive heatmap of immune microenvironment signatures, immune-checkpoint genes, and 22 types of infiltrated immune cells between Cluster1 and Cluster2 subtypes. **B** Heatmap of Cluster1 and Cluster2 subtypes for response of anti-CTLA-4 and anti-PD-1 in Chen et al. study. The results manifested that Cluster1 subtype could be more sensitive to the PD-1 inhibitor. **C** Heatmap of Cluster1 and Cluster2 subtypes for response of PD-1 in Prat A et al. study. The results indicated that Cluster1 subtype could have more chance to achieve complete response for anti-PD-1 therapy. **D** Heatmap of Cluster1 and Cluster2 subtypes for response of PD-1 in Hugo et al. study. The results indicated that Cluster1 subtype is more promising to achieve complete response for anti-PD-1 therapy. **E** Significant drug-disease scores for UM patients in the Connectivity Map. The top five potential drugs are labeled. **F** The risk heatmap of deep learning (DL) signature in TCGA-UVM cohort. **G** Kaplan–Meier curve of survival probability between high- and low-score of DL-signature in TCGA-UVM cohort. **H** Kaplan–Meier curve of survival probability between high- and low-score of DL-signature in HX cohort. **I** Time independent ROC curves and corresponding AUC values for DL-signature in TCGA-UVM cohort. **J** Time independent ROC curves and corresponding AUC values for DL-signature in HX cohort

Additionally, we exhibited a complete heatmap containing 22 types of immune cells, immune-associated biomarkers, and immune-checkpoint genes to investigate subtype-specific immunophenotypes (Fig. 5A). According to the Wilcoxon tests, the immune score, and ESTIMATE score were considerably enriched in the Cluster1 subtype, whereas the Cluster2 subtype has higher tumor purity the han Cluster1 subtype. Meanwhile, the Cluster1 subtype had high levels of immune-checkpoint gene expression. The CD8 T cells, ncells B cell, and CD4 memory resting T cells were heavily infiltrated in the Cluster1 subtype.

### Immunotherapy response and potential drugs prediction

The landscape of immune characteristics indicated that the Cluster1 subtype is an immune-hot subtype, therefore, we speculated that the Cluster1 subtype may be more promising to respond of immunotherapy. To prove our observation, SubMap analysis was employed to compare the expression patterns of the Cluster1/Cluster2 subtypes with previously published datasets of melanoma patients who accepted anti-CTLA-4 and/or anti-PD-1 therapies. Compared to anti-CTLA-4 therapy, we surprisingly discovered that the Cluster1 subtype was more respond to anti-PD-1 treatment (*p*-value = 0.00; Bonferroni corrected *p*-value = 0.01) (Fig. 5B). To validate this result, participants in Prat A et al. study, and Hugo et al. study who received anti-PD-1 treatment were divided into four subgroups (complete response: CR; partial response: PR; progressive disease: PD; stable disease: SD). Notably, we discovered that the Cluster1 subtype was more promising to achieve complete response for anti-PD-1 therapy regardless in Prat A et al. study (*p*-value = 0.04; Fig. 5C) and Hugo et al. study (*p*-value = 0.05; Fig. 5D). Moreover, to discover novel therapeutic drugs for UM, we compared gene expression patterns of 1288 drugs in the Connectivity Map and found that five potential drugs contained 4.5.dianilinophthalimide (score = -1), STOCK1N.35874 (score = -0.793), arachidonyltrifluoromethane (score = -0.093), TTNPB (score = -0.088), and W.13 (score = -0.034) were promising to treat UM patients (Fig. 5E).

### Construction of histopathologic DL-signature and gene-signature

**Fig. 6:**
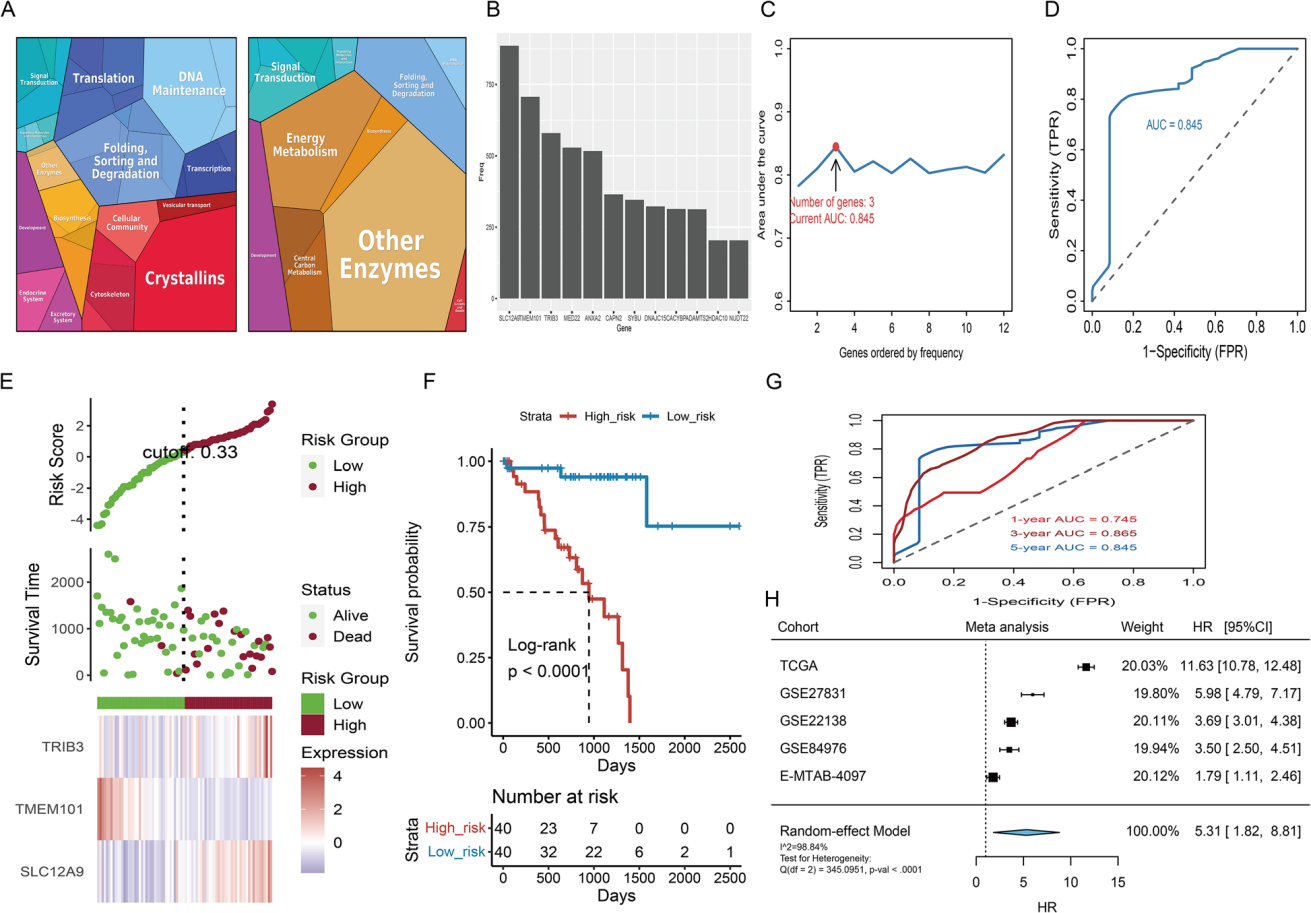
Discovery and validation of histopathologic gene-signature. **A** The KEGG enrichment of up- and down-regulated differentially expressed genes (DEGs). **B** The frequent distribution of histopathologic-related genes in 1000 Lasso-penalized selection and 12 genes were greater than 200 times. **C** AUC values for different histopathologic-related genes combination. When the combination is three, the AUC reaches the highest value (0.845). **D** The ROC curve for three gene combination. **E** The risk heatmap of UM patients in TCGA-UVM cohort. **F** Kaplan–Meier (K-M) curve of survival probability between high- and low-risk group in TCGA-UVM cohort. **G** Time dependent ROC (td-ROC) curves and corresponding AUC values in TCGA-UVM cohort. **H** Forest plot for meta-analysis of multiple UVM cohorts

**Fig. 7:**
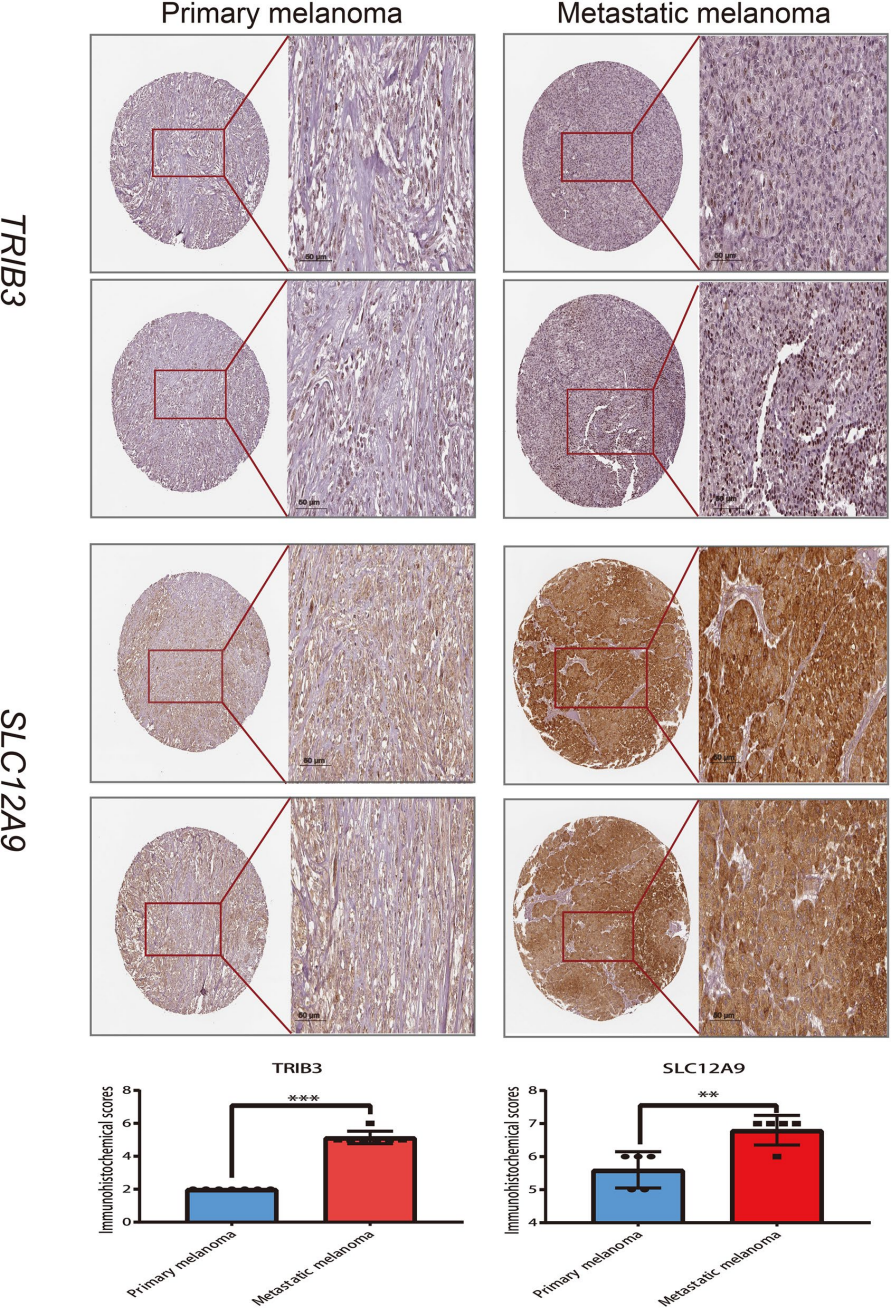
Immunohistochemical images and the box plots of the corresponding Immunohistochemical scores. (** represents *p* < 0.01, *** represents *p* < 0.001). The immunohistochemical scoring criteria considered staining intensity and the ratio of positive cells (Staining intensity: 0 for no, 1 for low, 2 for moderate, and 3 for strong. The ratio of positive cell s: < 25% is 1, 25%-50% is 2, 51%-75% is 3 and > 75% is 4)

Via multivariate Cox modeling, we constructed a DL-signature based on the relative score of 14 DL features in TCGA-UVM cohort. A comprehensive risk-heatmap was visualized to display the distribution of risk scores for patients in TCGA-UVM cohort, survival status, and the relative score of 14 DL features (Fig. 5F). Based on median of risk score, patients in TCGA-UVM cohort were split into two groups: high-risk (*n* = 40) and low-risk (*n* = 40). K-M curves uncovered that patients in high-risk have a poor survival with log-rank *p* < 0.0001 (Fig. 5G). The time-dependent ROC (td-ROC) showed that 1, 3 and 5 years of AUCs were 0.713, 0.860 and 0.953 (Fig. 5I). Furthermore, patients in HX cohort were accordingly classified into two groups: high-risk (*n* = 33) and low-risk (*n* = 34) and survival curves suggested that high-risk have a poor prognosis with log-rank *p* = 0.00062 (Fig. 5H). The td-ROC of 1, 3 and 5 years were 0.814, 0.829 and 0.846, respectively (Fig. 5J). To discovery the histopathologic gene-signature in UM, the samples of TCGA-UVM cohort (*n* = 80) were used as a training set, meanwhile GSE22138 (*n* = 63), GSE27831 (*n* = 29), GSE84976 (*n* = 28) and E-MTAB-4097 (*n* = 68) were treated as outside validation sets. Firstly, patients in the TCGA-UVM cohort, were classified into two subtypes: Cluster1 (*n* = 38) and Cluster2 (*n* = 42) based on histopathologic DL-signature; The differentially expressed analysis explored 142 DEGs in TCGA-UVM cohort (Table S4). The KEGG enrichment of DEGs was illustrated in Fig. 6A. Afterwards, univariate cox analysis revealed that 35 genes in DEGs were substantially linked with overall survival time in TCGA-UVM cohort (Table 3). Combining Lasso-penalized selection and AUC estimation, we found 12 histopathologic-related genes were greater than 200 (Fig. 6B). When the histopathologic-related signature contained 3 genes, the AUC of signature (TRIB3, TMEM101, and SLC12A9) was achieved the max value = 0.845 (Fig. 6C, D). Eventually, three genes were then utilized to build a gene-signature. The distribution of risk scores for patients in TCGA-UVM cohort, survival status, and genes expression level were visualized in a risk-heatmap (Fig. 6E). Based on median of risk score, patients in TCGA-UVM cohort were split into two groups: high-risk (*n* = 40) and low-risk (*n* = 40). K-M curves of high- and low-risk group revealed that high-risk group have a poor survival with log-rank *p*-value < 0.0001 (Fig. 6F). The td-ROC showed that 1, 3 and 5 years of AUCs were 0.745, 0.865 and 0.845 (Fig. 6G). In the validation sets (GSE22138, GSE27831, GSE84976 and E-MTAB-4097), these UM patients accordingly classified into low- and high-risk categories according to median cutoff. We discovered that UM patients in the low-risk group had a significantly longer survival probability than those in the high-risk group no matter in GSE22138 (Figure S1A; log-rank *p*-value < 0.0001), GSE27831 (Figure S1C; log-rank *p*-value = 0.0078), GSE84976 (Figure S1E log-rank *p*-value = 0.011), and E-MTAB-4097 (Figure S1G; log-rank *p*-value = 0.048). The AUC values of gene-signature were 0.600, 0.764, and 0.718, respectively, at 1, 3, and 5 years of survival time in the GSE22138 cohort (Figure S1B). In the GSE27831 cohort, the AUC values of gene-signature were 0.601, 0.732, and 0.565 at 1, 3 and 5 years respectively (Figure S1D). Moreover, the AUC values of gene-signature in the GSE84976 cohort were 0.717, 0.822, and 0.799, respectively at 1, 3 and 5 years (Figure S1F). In the E-MTAB-4097 cohort, the AUC values were 0.938, 0.706 and 0.663 at 1, 3 and 5 years respectively (Figure S1H). Ultimately, a meta-analysis was conducted to evaluate the use of risk scores in prognostic prediction. These four cohorts were included in a meta-analysis, which revealed that histopathologic-related gene signature was a risk factor influencing UM survival, with HR = 5.31 (95% CI:1.82 to 8.81) (Fig. 6H). To validate the results at the protein level, we performed immunohistochemistry of TRIB3 and SLC12A9 (risk genes) in metastatic and primary melanoma tissues. Compared to primary samples, the proteins of TRIB3 and SLC12A9 were highly expressed in metastatic melanoma (Fig. 7).

**Table 3:** Univariate cox analysis of 35 prognostic differentially expressed genes (DEGs) in TCGA-UVM cohort

features	univ_beta	univ_HR	univ_95% CI for HR	univ_p.value
SYBU	-0.37195	0.689392	0.506205–0.93887	0.018263
CYB5B	0.15665	1.16959	1.02022–1.34082	0.024629
UBE2Q1	0.112354	1.11891	1.00495–1.24579	0.040357
TOMM5	0.342533	1.40851	1.05422–1.88187	0.020496
PCBP2	-0.04234	0.958543	0.924196–0.994167	0.022957
C2orf72	-0.5483	0.577931	0.353942–0.943669	0.028399
NETO2	0.287774	1.33346	1.02362–1.73707	0.032923
DNAJB1	0.043162	1.04411	1.00245–1.08749	0.03773
DNAJC15	0.339647	1.40445	1.00364–1.96532	0.04757
CDK15	-1.20182	0.300646	0.096355–0.938072	0.038445
TUBB6	0.084527	1.0882	1.01949–1.16154	0.011083
RAN	0.026679	1.02704	1.00067–1.0541	0.04438
CRYAB	0.007802	1.00783	1.00176–1.01394	0.011358
TXNL4B	-0.37393	0.688024	0.47465–0.997317	0.048365
CACYBP	0.141074	1.15151	1.03932–1.27581	0.006986
CAPN2	0.071074	1.07366	1.00995–1.14139	0.022773
DEDD	0.202697	1.2247	1.01305–1.48057	0.036265
ZNF185	-0.46363	0.628998	0.464512–0.851729	0.002721
ANXA2	0.018306	1.01847	1.00856–1.02848	0.000243
VMAC	-0.73929	0.477454	0.290369–0.785077	0.003573
NOXA1	-0.36713	0.692717	0.535862–0.895486	0.005068
SLC12A9	0.568027	1.76478	1.37961–2.25748	6.14E-06
HDAC10	0.47998	1.61604	1.16999–2.23214	0.003583
TRIB3	0.110884	1.11727	1.03513–1.20592	0.004423
PCED1A	-0.11676	0.8898	0.806807–0.981329	0.019427
NUDT22	0.199332	1.22059	1.09065–1.36601	0.000519
ADAMTS2	0.18112	1.19856	1.08534–1.32359	0.000347
COX6A2	-0.07771	0.925233	0.870973–0.982872	0.011727
STK10	0.065302	1.06748	1.02017–1.11699	0.004753
ABHD12	0.028458	1.02887	1.00135–1.05714	0.039609
SH3TC1	0.350509	1.41979	1.06157–1.89889	0.018141
MRM1	0.149227	1.16094	1.05667–1.27549	0.001883
HPSE2	-0.98266	0.374316	0.162647–0.861452	0.020852
TMEM101	-0.05321	0.948186	0.916737–0.980713	0.00199
MED22	-0.51298	0.598711	0.437944–0.818494	0.001303

### Nomogram building and estimating

**Fig. 8:**
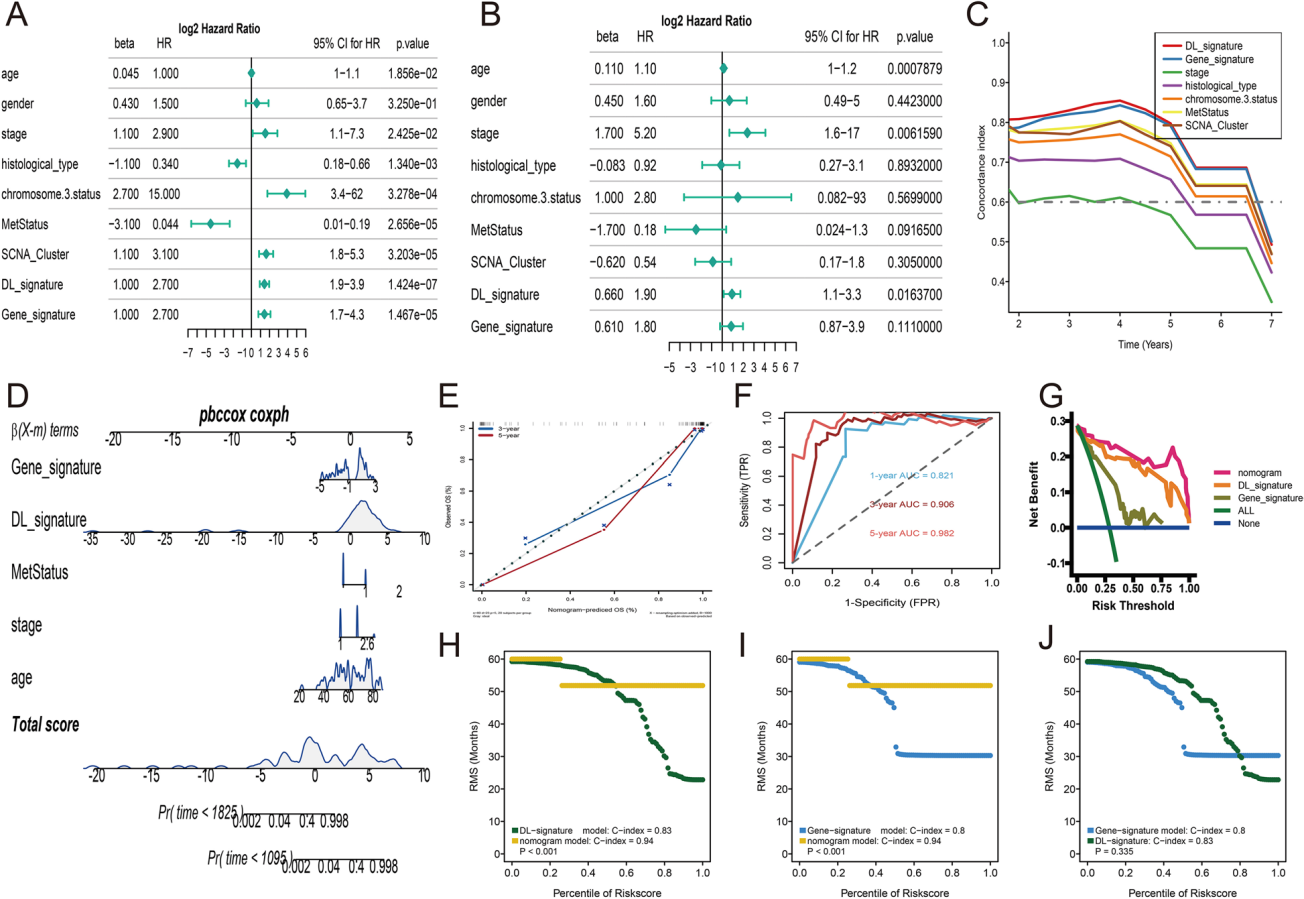
Construction of nomogram. **A** Forest plots of univariate Cox regression for histopathologic DL-signature, gene-signature, and clinical variables. **B** Forest plots of multivariate Cox regression for histopathologic DL-signature, gene-signature, and clinical variables. **C** Time dependent C-index of DL-signature, gene-signature, and clinical variables (tumor stage, histological type, metastasis status, chromosome 3 status and SCNA_Cluster). **D** Nomogram included DL-signature, gene-signature, age, tumor stage and metastasis status which predicts the 3-, and 5-year of overall survival time for patients TCGA-UVM cohort. **E** Calibration curves of the nomogram for the estimation of 3-, and 5-year of overall survival rates. **F** Time dependent ROC of nomogram. **G** The decision curves of nomogram, DL-signature, and gene-signature. **H** RMS curves for DL-signature and nomogram. **I** RMS curves for gene-signature and nomogram. **J** RMS curves for DL-signature and gene-signature. Each point represents the RMS time of corresponding DL-signature, gene-signature and nomogram scores

To provide a comprehensive and accurate approach for prognosis prediction, a nomogram was created using histopathologic DL-signature, gene-signature and clinical variables from patients in the TCGA-UVM cohort. To begin, we applied univariate and multivariate Cox analyses to contrast the prognostic performance of DL-signature, gene-signature, and other clinical variables. We discovered that age, tumor stage, histology type, chromosome 3 status, metastasis status, SCNA cluster, DL-signature, and gene-signature were all correlated significantly with overall survival of UM in univariate regressions (Fig. 8A), while only age, tumor stage, and DL-signature were significantly associated with overall survival of UM in multivariate regressions (Fig. 8B). Besides, to compare the prognostic ability of the DL-signature, and gene-signature with clinical variables, time-dependent C-index curves uncovered that the DL-signature was similar to the gene-signature and obviously higher than other clinical variables such as tumor stage and chromosome 3 status (Fig. 8C). Thus, the DL-signature, gene-signature, age, tumor stage and metastasis status (*p*-value >  = 0.1 in multivariate regressions) were integrally merged into a nomogram model which can forecast the possibility of overall survival at three and five years (Fig. 8D). The calibration curve of the nomogram at 3- and 5-year manifested a better consistency between the prediction and actual observations (Fig. 8E). The td-ROC curve to estimate the accuracy of the nomogram model and found that the 1-, 3-, and 5-year of AUCs had a higher accuracy (> 0.80) (Fig. 8F). In the decision curve, we observed that our nomogram model can achieve higher net benefits than DL-signature, and gene-signature (Fig. 8G). Besides, we conducted a restricted mean survival (RMS) analysis to assess the prognostic value of DL-signature, gene-signature, and nomogram model. The RMS curve uncovered that the nomogram had a larger slope than the DL-signature (Fig. 8H), and gene-signature (Fig. 8I), which indicated a superior performance of the nomogram model for survival prediction. However, there is no significant difference between DL-signature and gene-signature in RMS curve estimation (Fig. 8J).

## Discussion

H&E-stained histopathological images can give valuable information for clinical decision-making in a wide range of malignancies [30]. However, due to the requirement of experience of pathologists, immunohistochemical or extra genetic testing restricted its availability to the general public [31]. To the best of our knowledge, our work is the first time to show that a DL model can be used to predict the vital status of UM patients. In this work, the results show that our developed DL model can achieve a high accuracy of 90% for patches and WSIs prediction. It is widely available in clinical practice, allowing any patient with a pathological diagnosis to obtain a prognosis estimation. Besides, we also investigated the interpretability of the model in terms of genome and transcriptome connection, providing a bioinformatic interpretation for our model.

In principle, classification validity is beneficial in predicting the clinical importance of genotype in terms of therapy responsiveness. As a consequence, our DL features classified UM patients into subtypes (Cluster1/Cluster2) with distinct clinical outcomes, tumor mutation, immune microenvironment, and molecular pathways. Compared to Cluster1, UM patients in Cluster2 have a good prognosis. Regarding tumor mutation, we discovered that BAP1 was more frequently mutated in the Cluster1 subtype, which was consistent with prior evidence suggesting BAP1 mutations enhance the possibility of UM patients developing metastases [32]. Furthermore, mutations in EIF1AX and SF3B1 were more common in the Cluster2 subtype, which has been shown to have protective roles in the prognosis of UVM patients [33, 34]. In molecular pathways, we observed that 10 cancer Hallmark pathways including glycolysis, hypoxia, IL2-STAT5-signaling, ILl6-JAK-STAT5 signaling, MTORC1 signaling, notch signaling, peroxisome, reactive oxygen species pathway, spermatogenesis, and unfolded protein response were actively enriched in Cluster1 subtype. More intriguingly, these pathways are reported to be associated with malignant transformation [35–37]. For example, the previous findings in melanoma revealed that unfolded protein response is positively linked with tumor development, size, and patient prognosis [38, 39]. Moreover, we noticed that a high infiltration of the immune microenvironment existed in the Cluster1 subtype, as well as closely associated immune-checkpoint molecular such as PD-1, CTLA-4, LAG3, and PDCD1. Astonishingly, growing evidence shows that increased lymphocytic immune cell infiltration, such as CD8 + T and CD4 + T cells, are poor predictors of UM patient prognosis [40–42]. Taking all findings into account, it's clear why DL features classified as Cluster1 subtype have a poorer prognosis than the Cluster2 subtype.

In addition, we discovered that the Cluster1 subtype has a higher expression level of immune-checkpoint molecular and is more likely to react to immunotherapy. Compared to anti-CTLA-4 therapy, UM patients in the Cluster1 subtype were more sensitive to anti-PD-1 treatment and more likely to achieve a complete response. In fact, just a small proportion of UM patients in clinical studies are responding to immunotherapies. Therefore, exploration of suitable drugs for UM therapy is thus required. Via Connectivity Map analysis, we found that five potential drugs contained 4.5. dianilinophthalimide, STOCK1N.35874, arachidonyltrifluoromethane, TTNPB, and W.13 were promising to treat UM patients. 4.5.dianilinophthalimide is a selective inhibitor of the epidermal growth factor receptor signal transduction pathway which is essential for the growth and metastasis of various cancer cells [43–45]. The cytosolic phospholipase A2 inhibitor (arachidonyltrifluoromethane) can hinder many critical pathways involved in the development of recurrent resistant cancer [46]. The pharmacological mechanisms of these drugs in UM patients can be used as a direction for future research.

It would be a ground-breaking way to characterize gene expression patterns for DL feature-related phenotypes to create patient-specific personalized treatments. We next investigated the DEGs within these subtypes and discovered that these histopathological-related genes were positively linked with genetic information processing, cellular processes, and metabolism. Based on these DEGs, we established a prognostic gene-signature in the TCGA-UVM cohort and verified its prognostic values in several independent datasets. The gene-signature contained three genes, including TRIB3, TMEM101, and SLC12A9, of which some have been reported to be correlated to melanoma and other tumors. For example, via suppressing autophagy and ubiquitin–proteasome degradation processes, TRIB3 can promote melanoma progression [47]. The transmembrane protein TMEM101 has been demonstrated to stimulate the NF-kappa-beta signaling pathways. Medha et al. reported that methylation of the TMEM101 promoter works as a potential predictive biomarker for breast cancer [48]. As a result, it is plausible to anticipate that our discovered gene-signature can be used as a predictive biomarker in a future clinical study. When compared to conventional clinical characteristics, our constructed DL-signature and gene-signature outperformed the clinical features (eg. Tumor stage, histology type, chromosome 3 status, and SCNA_cluster). Importantly, univariate and multivariate Cox regression demonstrated that our DL-signature may be used as an independent prognostic predictor in UM to offer a fairly accurate prediction of overall survival. Finally, a systemic nomogram combining DL-signature and gene-signature was proven to possess high predictive power and guide clinicians on optimal treatment approaches to increase the practical application value of the histopathological-related signature.

## Conclusions

Overall, we created a deep learning model for vital status prediction based on histopathological images. Using these pictures, it is now feasible to assess the prognosis of UM patients more than many previous works. According to histopathological DL features, we found out two subgroups that may be in favor of immunotherapy and chemotherapy. Furthermore, we developed a systemic nomogram that combines DL-signature and gene-signature to give a more straightforward and reliable prognosis for UM patients in treatment and management.

## Supplementary Information

**Additional file 1:** **Table S1.** Baseline features of three cohorts.

**Additional file 2:** **Table S2.** 379 deep learning (DL) features from histogram of patches likelihood.

**Additional file 3:** **Table S3.** The detail parameters for assessment of seven machine learning models.

**Additional file 4:** **Table S4.** The differentially expressed genes (DEGs) in TCGA-UVM cohort.

**Additional file 5:** **Figure S1.** A: K-M curve of survival probability in GSE22138 cohort. B: td-ROC curves and corresponding AUC values in GSE22138 cohort. C: K-M curve of survival probability in GSE27831 cohort. D: td-ROC curves and corresponding AUC values in GSE27831 cohort. E: K-M curve of survival probability in GSE84976 cohort. F: td-ROC curves and corresponding AUC values in GSE84976 cohort. G: K-M curve of survival probability in E-MTAB-4097cohort. H: td-ROC curves and corresponding AUC values in E-MTAB-4097 cohort.

## Data Availability

The datasets used in the current study are available from the corresponding author on reasonable request.

## Abbreviations

TCGA-UVM: Uveal melanoma of TCGA data
GEO: Gene Expression Omniniub
DL: Deep learning
UM: Uveal melanoma
WSI: Whole-slide image
TMB: Mutation burden
MSI: Microsatellite instability
AUC: The area under the curve
td-ROC: The time-dependent ROC
RMS: Restricted mean survival
