# Supplementary figures and images for: Deep learning classification of uveal melanoma based on histopathological images and identification of a novel indicator for prognosis of patients
Source: Biol Proced Online. 2023 Jun 2;25:15. doi: 10.1186/s12575-023-00207-0 (PMC10239131; doi:10.1186/s12575-023-00207-0)

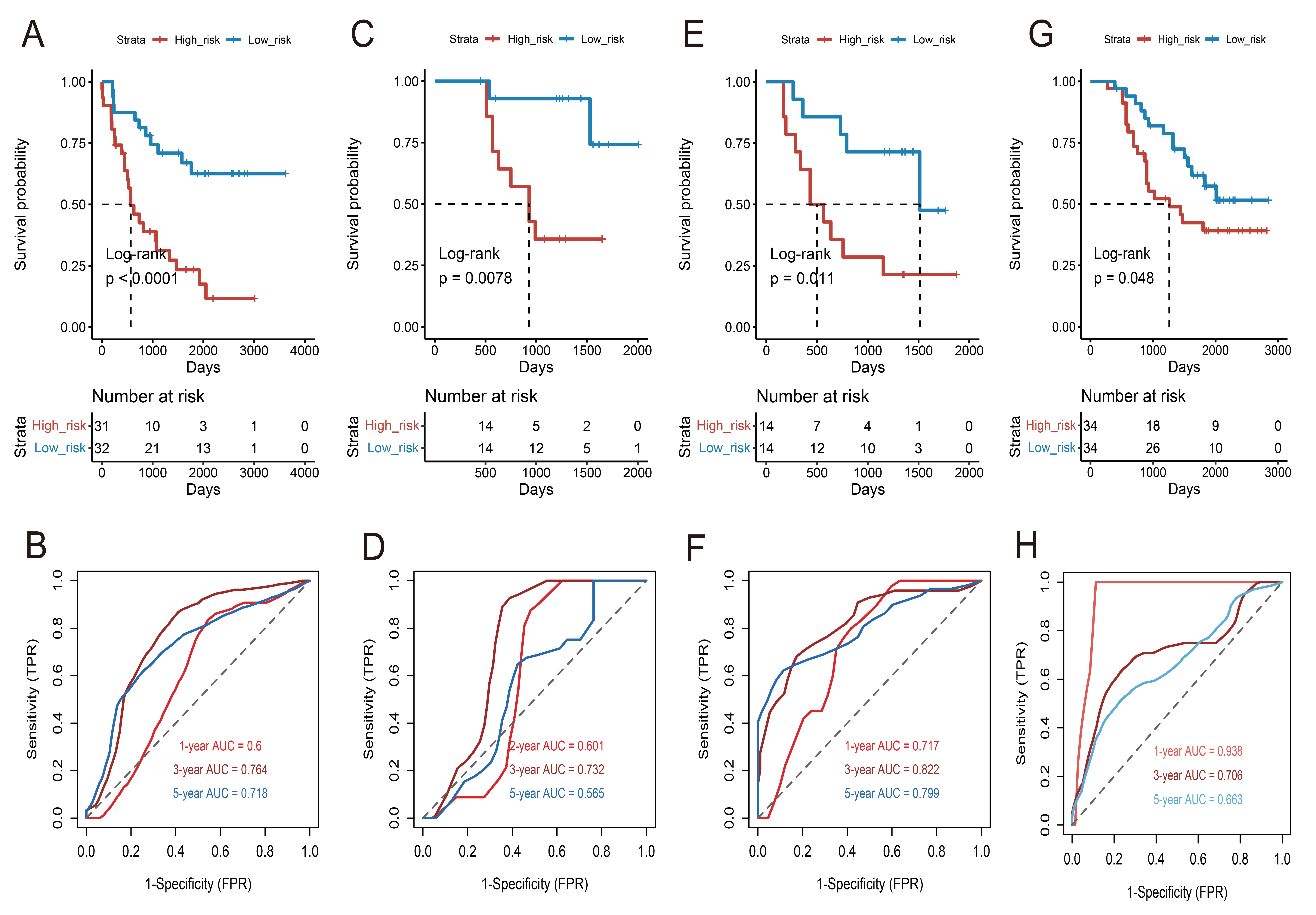

Supplement: Supplementary file 5 — Additional file 5: Figure S1. A: K-M curve of survival probability in GSE22138 cohort. B: td-ROC curves and corresponding AUC values in GSE22138 cohort. C: K-M curve of survival probability in GSE27831 cohort. D: td-ROC curves and corresponding AUC values in GSE27831 cohort. E: K-M curve of survival probability in GSE84976 cohort. F: td-ROC curves and corresponding AUC values in GSE84976 cohort. G: K-M curve of survival probability in E-MTAB-4097cohort. H: td-ROC curves and corresponding AUC values in E-MTAB-4097 cohort. [file 12575_2023_207_MOESM5_ESM.jpg]
